# New Insights into the Pro-Inflammatory Activities of Ang1 on Neutrophils: Induction of MIP-1β Synthesis and Release

**DOI:** 10.1371/journal.pone.0163140

**Published:** 2016-09-15

**Authors:** Elizabeth Dumas, Paul-Eduard Neagoe, Patrick P. McDonald, Michel White, Martin G. Sirois

**Affiliations:** 1 Research center, Montreal Heart Institute, Montreal (Quebec), Canada; 2 Departments of pharmacology, Faculty of medicine, Université de Montréal, Montreal (Quebec), Canada; 3 Pulmonary Division/Research, Faculty of Medicine, Université de Sherbrooke, Sherbrooke (Quebec), Canada; 4 Departments of medicine, Faculty of medicine, Université de Montréal, Montreal (Quebec), Canada; Universidade Federal do Rio de Janeiro, BRAZIL

## Abstract

We reported the expression of angiopoietin Tie2 receptor on human neutrophils and the capacity of angiopoietins (Ang1 and Ang2) to induce pro-inflammatory activities, such as platelet-activating factor synthesis, β_2_-integrin activation and neutrophil migration. Recently, we observed differential effects between both angiopoietins, namely, the capacity of Ang1, but not Ang2, to promote rapid interleukin-8 synthesis and release, as well as neutrophil viability. Herein, we addressed whether Ang1 and/or Ang2 could modulate the synthesis and release of macrophage inflammatory protein-1β (MIP-1β) by neutrophils. Neutrophils were isolated from blood of healthy volunteers; intracellular and extracellular MIP-1β protein concentrations were assessed by ELISA. After 24 hours, the basal intracellular and extracellular MIP-1β protein concentrations were ≈500 and 100 pg/10^6^ neutrophils, respectively. Treatment with Ang1 (10 nM) increased neutrophil intracellular and extracellular MIP-1β concentrations by 310 and 388% respectively. Pretreatment with PI3K (LY294002), p38 MAPK (SB203580) and MEK (U0126) inhibitors completely inhibited Ang1-mediated increase of MIP-1β intracellular and extracellular protein levels. Pretreatment with NF-κB complex inhibitors, namely Bay11-7085 and IKK inhibitor VII or with a transcription inhibitor (actinomycin D) and protein synthesis inhibitor (cycloheximide), did also abrogate Ang1-mediated increase of MIP-1β intracellular and extracellular protein levels. We validated by RT-qPCR analyses the effect of Ang1 on the induction of MIP-1β mRNA levels. Our study is the first one to report Ang1 capacity to induce MIP-1β gene expression, protein synthesis and release from neutrophils, and that these effects are mediated by PI3K, p38 MAPK and MEK activation and downstream NF-κB activation.

## Introduction

Angiogenesis is a critical process to many biological conditions, such as physiological somatic growth and vascular repair, but also to pathological cancer and rheumatoid arthritis [1]. What distinguishes pathological from physiological angiogenesis is the presence of inflammation, which is required to initiate angiogenesis in a pathological environment and its contribution to the intensification of chronic inflammatory status [2]. Inflammatory properties of angiogenic factors may therefore play a critical role in establishing and supporting pathological angiogenesis, thus making the characterization of those inflammatory effects essential to better understand the mechanisms leading to the progression of many chronic diseases and to identify new therapeutic targets to block pathological angiogenesis.

Three majors growth factors have been described for their participation in the angiogenic process: namely, the vascular endothelial growth factor (VEGF), which induces the formation of new blood vessels; angiopoietin-1 (Ang1), which contributes to the stabilization of the neovessels; and angiopoietin-2 (Ang2), which disrupts pre-existing vasculature [3]. In addition to their participation to pro-angiogenic activities, we and other groups reported that these growth factors can also induce several pro-inflammatory activities mainly in endothelial cells (ECs) but also in leukocytes. For instance, VEGF increases vascular permeability through the induction of nitric oxide (NO) and platelet activating factor (PAF) synthesis by ECs [4, 5], and also facilitate leukocyte adhesion and transmigration through the translocation and expression of endothelial adhesion molecules, namely P-selectin, E-selectin, intercellular adhesion molecule-1 (ICAM-1) and vascular cell adhesion molecule-1 (VCAM-1) [6, 7]. Moreover, it has been reported that both angiopoietins induce many inflammatory activities in ECs, such as modulating EC survival and potentiating VEGF effects in a murine model of neovascularization [8, 9]. In our laboratory, we demonstrated that both Ang1 and Ang2 induce, in a Tie2-dependent manner, endothelial PAF synthesis, endothelial P-selectin translocation and neutrophil adhesion onto ECs [10, 11]. As opposed to ECs, which express both Tie1 and Tie2 receptors, we reported that only Tie2 receptor is expressed on neutrophils [11]. We and other groups have also reported that both angiopoietins in a Tie2-dependent manner are capable to recruit neutrophils and eosinophils as well as to promote neutrophil adhesion onto human extracellular matrix [11–14]. In addition, both Ang1 and Ang2 induce neutrophil migration and potentiate interleukin-8 (IL-8) chemotactic activity [12, 14]. More recently, we also reported that Ang1 and Ang2 can have differential pro-inflammatory effects on neutrophils; for example, Ang1 increases neutrophil viability in a Tie2-dependent manner and through the release of IL-8 synthesis and release. Ang1 has also the capacity to induce IL-1β synthesis and IL-1RA release, while Ang2 has no such effects [15–17].

Neutrophils are the first immune cells to be recruited at inflammatory sites and their principal roles are to secrete lytic enzymes to induce cellular toxicity and to prepare the immune response upon release of different cytokines to attract specific leukocyte populations. Neutrophils have the capacity to secrete numerous chemokines such as IL-1β, IL-8, GROα, IP-10 (interferon-gamma-inducible protein-10), MIG (monokine induced by gamma interferon), macrophage inflammatory protein-1α (MIP-1α) and MIP-1β [18]. MIP-1β (CCL4), who acts upon interaction with CCR5 receptor, is expressed in many leukocytes including macrophages, dendritic cells, lymphocytes and neutrophils [19]. It has been reported that upon its release by the neutrophils, MIP-1β is a crucial chemotactic mediator for monocytes/macrophages (MΦ) recruitment [20]. In addition, MIP-1β can also attract other immune cells, namely T lymphocytes, dendritic cells and natural killer (NK) cells [19]. Therefore, MIP-1β released by neutrophils allows the progression of inflammatory processes by recruiting other leukocytes into inflamed tissues, leading either to inflammation resolution through efferocytosis by macrophages or to the development of chronic inflammation. In view of our finding that Ang1 can induce cytokine synthesis and release in neutrophils [15, 17] and because MIP-1β is known to be an important mediator of MΦ influx [20], we assessed the effect of Ang1 and Ang2 on MIP-1β synthesis and release from neutrophils, as well as the intracellular mechanisms involved.

## Materials and Methods

### Neutrophil isolation and purification

The study and consent procedure was in accordance with the Declaration of Helsinki and approved by the Montreal Heart Institute's ethical committee (Montreal, QC, Canada; ethics No. ICM#01–069 and ICM#12–1374). All of the subjects provided written informed consent to the experimental protocol before participating in the study. Neutrophils were isolated as described previously [11, 14]. Neutrophils were resuspended in RPMI-1640 medium (Cambrex BioScience, Walkersville, MD, USA) supplemented with 25 mM Hepes (N-2-hydroxyethylpiperazine-N’-2-ethanesulfonic acid) (Sigma, Oakville, ON, Canada), 1% penicillin/streptomycin/Glutamax (GIBCO, Grand Island, NY, USA) and 5% fetal bovine serum (PAA Laboratories, Etobicoke, ON, Canada), and termed RPMI (for complete RPMI-1640 solution). Contamination of isolated neutrophil suspension with peripheral blood mononuclear cells was less than 0.1% as determined by morphological analysis and flow cytometry, viability was found to be greater than 98%, as assessed by Trypan blue dye exclusion assay [17].

### Measurement of intracellular and secreted MIP-1β by ELISA

Neutrophils (5x10^6^/ml) were incubated at 37°C for different periods of time (0–24 hours) with different agonists (bacterial lipopolysaccharide; LPS; Escherichia coli) (Sigma), Ang1 and Ang2 (R&D Systems, Minneapolis, MN, USA) in RPMI. Supernatants were collected by centrifugation at 900g for 7 minutes and neutrophil pellets were suspended in lysis buffer (RPMI containing 1% Triton (J.T. Baker; Phillipsburg, NJ, USA) and protease inhibitor (Pefabloc, 1 mM; Sigma)) to collect intracellular neutrophil content. Cells were vortexed for 30 seconds, centrifuged at 15 000g for 7 minutes at 4°C and sonicated as described previously [21]. Samples were stored at -80°C for further quantification of MIP-1β by ELISA development kits (R&D Systems) accordingly to manufacturer’s instructions. In another set of experiments, neutrophils were pretreated with either a mRNA transcription inhibitor (Actinomycin D; Sigma), a protein synthesis inhibitor (Cycloheximide; Calbiochem, La Jolla, CA, USA) or inhibitors of p38 MAPK (SB203580; Calbiochem), MEK (U0126; Millipore, Billerica, MA, USA), PI3K (LY294002; Cayman Chemical, Ann Arbor, MI, USA) and NF-κB pathway (IKK inhibitor VII; Calbiochem and Bay11-7085; Santa Cruz Biotechnology, Dallas, TX, USA) for 5 minutes prior to agonist stimulation for 24 hours as described above. Supernatants and intracellular neutrophil protein contents were then collected and stored at -80°C for MIP-1β quantification as aforementioned.

### Phosphorylation analyses

Neutrophils (1x10^7^/ml) were pretreated with selected inhibitors (control vehicle, SB203580, U0126, LY294002, IKK inhibitor VII and Bay11-7085) for 5 minutes prior to agonist stimulation with LPS and Ang1 at 37°C for 0.5–60 minutes as described above. At the end of each incubation, neutrophils were centrifuged at 10 000g for 30 seconds at 4°C and the supernatant discarded. Neutrophil pellets were suspended in ice cold PBS, supplemented with diisopropyl fluorophosphate (DFP; 2 mM; Santa Cruz Biotechnology), phosphatase inhibitors (10 mM NaF; Merck KGaA, Darmstadt, Germany, 1 mM Na_3_VO_4_; Sigma, 10 mM Na_4_P_2_O_7_; Sigma) and antiprotease cocktail (dithiothreitol (DTT; Amresco, Solon, OH, USA), 1 mmol/L PMSF, 1 mmol/L 4-(2-amino ethyl) benzene sulfonyl fluoride (AEBSF), and 10 μg/mL each of aprotinin and leupeptin; all from Sigma). Then, boiling 2x sample buffer was added directly to cell pellets, which were briefly vortexed and incubated at 96°C for an additional 5 minutes. These samples were then sonicated to disrupt chromatin and stored at -20°C prior to analysis. Samples were electrophoresed, transferred onto polyvinylidene fluoride (PVDF) membrane, and processed for immunoblot analysis as described previously [14] with specific antibodies for phospho-p38, phospho-ERK1/2, phospho-Akt, Akt, phospho-p65 and p65 (NF-κB) (Cell Signaling Technology, Danvers, MA, USA), and for p38 MAPK, ERK1/2 and anti-rabbit IgG-HRP (Santa Cruz Biotechnology).

### Real time quantitative transcriptase-polymerase chain reaction (RT-qPCR) analyses

Neutrophils (5x10^6^/ml) were stimulated (0–24 hours) as described for ELISA analyses. Total RNA was isolated by using RNeasy extraction kit (Bio-Tek, Norcross, GA, USA). Seventy-five (75) ng of RNA was reverse transcribed using random hexamers and the Moloney murine leukemia virus (MMLV) reverse transcriptase (Invitrogen, Burlington, ON, Canada) as described by the manufacturer. Primers were used to amplify a 667-base pair (bp) fragment of MIP-1β cDNA (GenBank no. MIM182284; forward: 50-GCTAGTAGCTGCCTTCTGCTCTCC-30, and reverse 50-CAGTTCCAGGTCATACACGTACTCC-30) and a 93-bp fragment of β-microglobulin cDNA (GenBank no. MIM109700; forward: 50-TAGCTGTGCTCGCGCTACTC-30, and reverse: 50-TTCCATTCTCTGCTGGATGACG-30). The expression level of MIP-1β was determined using a Brilliant SYBR Green qPCR master mix (Invitrogen) and Mx3000P real-time PCR system (Agilent Technologies, Cedar Creek, TX). To confirm the specificity of the PCR products, the melting profile of each sample was determined by heating from 55°C to 95°C while measuring the fluorescence emitted. Analysis of the melting curve showed that each pair of primers amplified a single product. Each run consisted of an initial denaturation time of 10 minutes at 95°C and 40 cycles at 95°C for 30 seconds, 55°C for 60 seconds, and 72°C for 60 seconds.

### Statistical analyses

Results are presented as the mean ± SEM from at least three (3) independent experiments, and each experiment is deriving from independent donors. Statistical comparisons were made by analysis of variance (ANOVA) followed by a Bonferroni *t*-test. Results were considered significantly different at *p* values less than 0.05.

## Results

### Ang1 induces MIP-1β synthesis and release from human neutrophils

We reported the capacity of Ang1 to induce IL-8 and IL-1β synthesis from human neutrophils and the release of IL-8 and IL-1RA, while Ang2 had no such effects [15, 17]. To further evaluate the potential of angiopoietins to modulate the synthesis and release of other inflammatory cytokines, we sought to determine whether Ang1 and/or Ang2 could induce the synthesis and release of MIP-1β in neutrophils.

**Fig 1:**
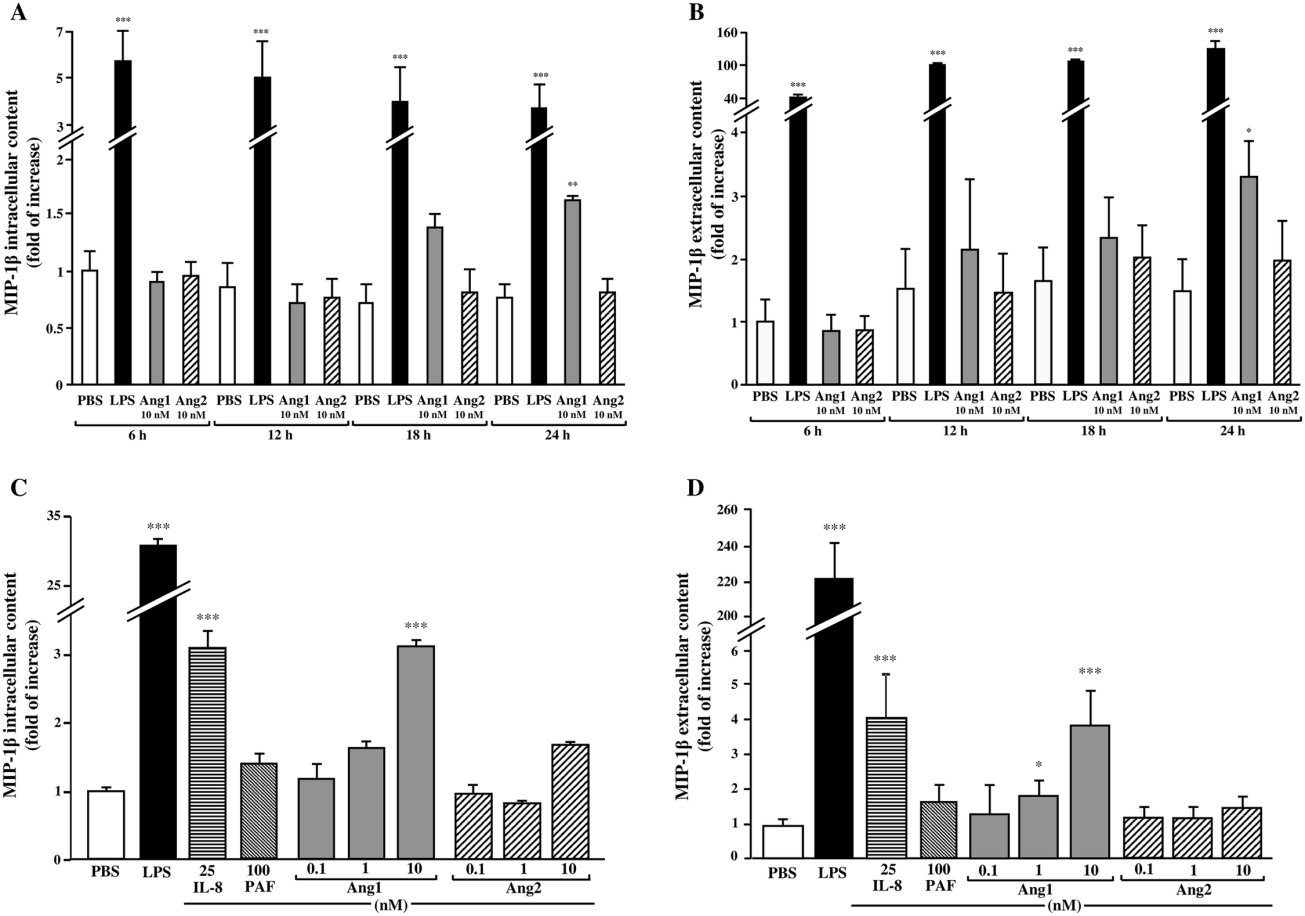
Effect of angiopoietins on MIP-1β synthesis and release in a time- and concentration-dependent manner. Human neutrophils were incubated with LPS (100 ng/ml; positive control), Ang1 or Ang2 (10 nM) for 6 to 24 hours at 37°C (A and B). Supernatants and cell lysates were collected and MIP-1β protein synthesis (A) and release (B) were evaluated by ELISA. In another set of experiments, neutrophils were incubated with LPS (100 ng/ml), IL-8 (25 nM), PAF (100 nM), Ang1 or Ang2 (0.1–10 nM) for 24 hours at 37°C and MIP-1β protein synthesis (C) and release (D) were evaluated. *p<0.05; **p<0.01 and ***p<0.001 as compared to PBS-treated cells (n = 3–7 experiments).

In a first series of experiments, we assessed the capacity of both angiopoietins to induce MIP-1β synthesis and release from neutrophils in a time- (0–24 hours) and concentration- (0.1–10 nM) dependent manner (Fig 1). Neutrophils were treated with PBS (control vehicle), LPS (100 ng/ml; positive control)[22], Ang1 or Ang2 (10 nM) for 0, 2, 4, 6, 12, 18 or 24 hours. Supernatants and cell lysates were collected for MIP-1β protein quantification by ELISA. Immediately upon neutrophil isolation (0 hour), the intracellular MIP-1β protein content was undetectable in naïve neutrophils, while at 2 and 4 hours post-isolation, intracellular MIP-1β concentrations in PBS-treated neutrophils were approximately 130 and 180 pg/10^6^ neutrophils (data not shown). The basal release of MIP-1β (extracellular content) was not detectable immediately upon neutrophil isolation while at 2 and 4 hour post-isolation the extracellular MIP-1β protein content remained below the minimal detectable dose (MDD) certified by the manufacturer (<15.6 pg/ml; data not shown). Thus, we are reporting the data from 6 to 24 hour post-stimulation. The basal (PBS-treated) levels of MIP-1β intracellular and extracellular content at 6 hours were ≈500 and 100 pg/10^6^ neutrophils, respectively and did not change significantly over time (from 6 to 24 hours). Since the MIP-1β intracellular and extracellular content fluctuate from one donor to another, data for all figures are thus reported as relative fold of increase of MIP-1β protein content as compared to PBS-treated neutrophils, which is set to 1. Treatment with LPS significantly increased MIP-1β synthesis at every time-point and plateaued after 6 hours of stimulation as compared to PBS-treated cells, the protein intracellular content increased by 5.7-fold (from 546 to 3117 pg/10^6^ neutrophils) (Fig 1A). Treatment with Ang1 for up to 12 hours did not modulate significantly the intracellular MIP-1β protein concentration. However, upon 18 hours of stimulation, Ang1 provided a trend to increase MIP-1β intracellular content (p = 0.073), which became significant at 24 hour post-stimulation (2.1-fold increase; from 475 to 998 pg/10^6^ neutrophils) (Fig 1A). Treatment with Ang2 did not promote any significant increase of intracellular MIP-1β protein content at any time-point (Fig 1A). We then assess whether synthesized MIP-1β was released by the neutrophils upon stimulation by corresponding agonists. Treatment with Ang1 significantly increase MIP-1β release at 24 hour (2.2-fold increase of extracellular content as compared to PBS-treated neutrophils; ranging from 111 to 245 pg/10^6^ neutrophils) while no significant difference on MIP-1β protein extracellular content was observed at earlier time-points (Fig 1B). Treatment with Ang2, up to 24 hours did not modulate significantly the extracellular MIP-1β protein content (Fig 1B). As previously reported [22], LPS induced MIP-1β release from neutrophils at each time-point (nearly maximal within 6 hours post-stimulation; 69-fold increase; ranging from 113 to 7800 pg/10^6^ neutrophils), and being maximal at 24 hours (Fig 1B). Therefore, all subsequent studies related to MIP-1β intracellular and extracellular contents were addressed at 24-hour post-stimulation.

In another set of experiments, we assessed in a concentration-dependent manner, the capacity of Ang1 and Ang2 to induce MIP-1β synthesis and release. Treatment with Ang1 (1 and 10 nM) increased MIP-1β intracellular (1.6- and 3.2-fold respectively) and extracellular (1.9- and 3.9-fold respectively) protein content, while Ang2 had no significant effect (Fig 1C and 1D). MIP-1β synthesis and release was not evaluated at higher concentrations of Ang1 and Ang2 since 10 nM is the maximal concentration also used by other groups for studying angiopoietin activities [7, 23]. At lower concentration (0.1 nM), both Ang1 and Ang2 did not modulate MIP-1β intracellular and extracellular content (Fig 1C and 1D). Since Ang1 can prolong neutrophil viability at 24 hour post-stimulation, we also assessed if other agonists known for their capacity to prolong neutrophil viability (LPS, IL-8 and PAF) [22, 24, 25] were capable to support MIP-1β synthesis and release at 24 hours post-stimulation. Treatment with LPS (100 ng/ml) significantly increased the intracellular and extracellular MIP-1β levels by 31- and 218-fold respectively. Treatment with IL-8 (25 nM) also significantly increased intracellular and extracellular MIP-1β levels by 3.1 and 4.1-fold respectively, while PAF (100 nM) had no significant effect (Fig 1C and 1D).

### Ang1-mediated MIP-1β synthesis and release: Intracellular cell signaling pathways

**Fig 2:**
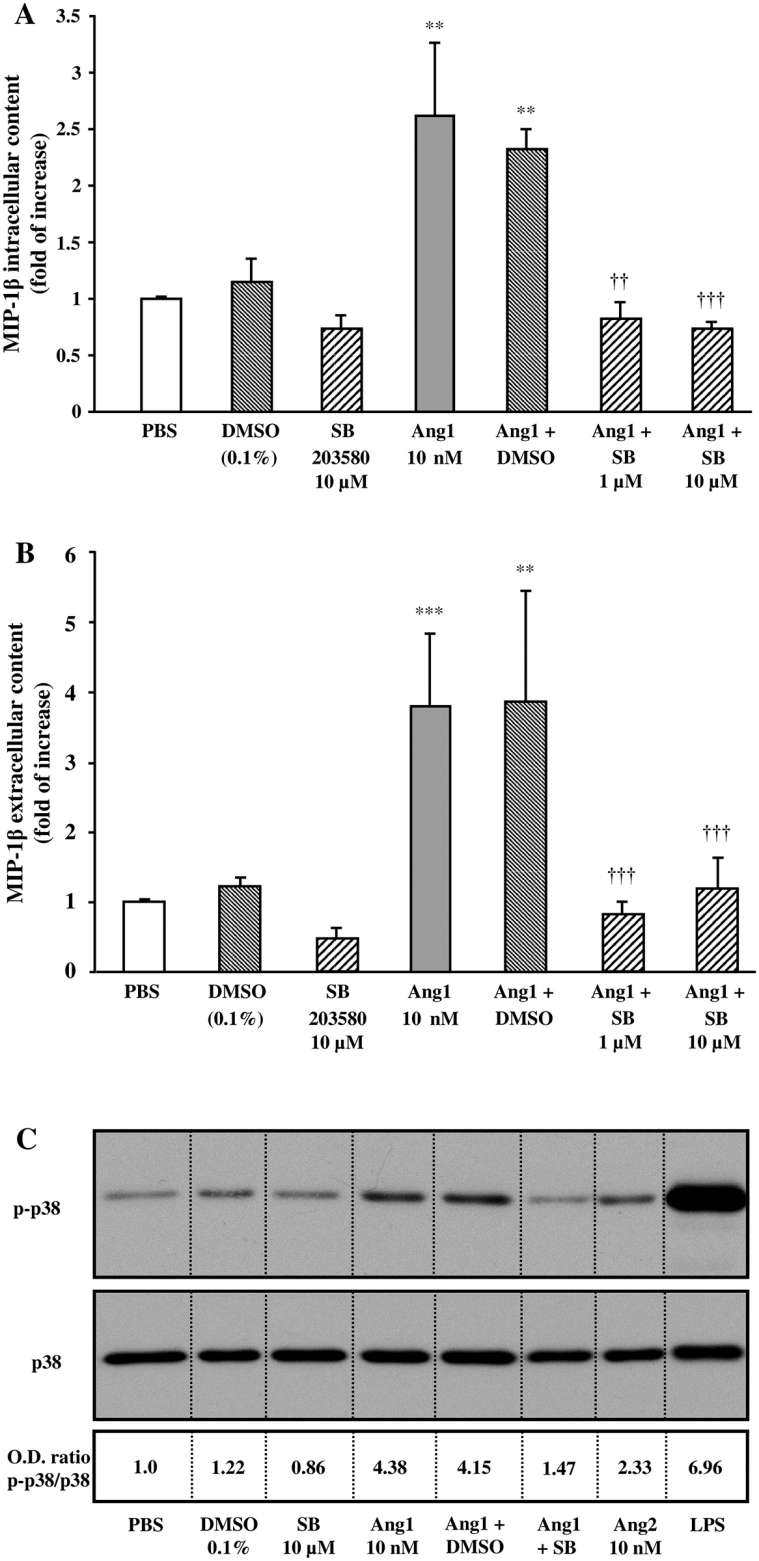
Effect of p38 MAPK pathway on Ang1-induced MIP-1β synthesis and release in neutrophils. Neutrophils were pretreated with or without SB203580 (1 or 10 μM) or with DMSO (0.1%; control vehicle) for 5 minutes. Then, neutrophils were incubated with or without Ang1 (10 nM) for 24 hours at 37°C. MIP-1β synthesis (A) and release (B) were evaluated by ELISA. In another set of experiments, neutrophils were stimulated with aforementioned inhibitors ± Ang1 for 2.5 minutes at 37°C; treatment with Ang2 (10 nM) and LPS (100 ng/ml) were added as comparative controls. Cell lysates were collected for Western blot analyses of p38MAPK phosphorylation and total p38MAPK protein (C). The image presented is a representative figure of at least three independent experiments. **p<0.01 and ***p<0.001 as compared to PBS-treated cells; ^††^p<0.01 and ^†††^p<0.001 as compared to Ang1-treated cells (Fig 2A and B; n = 3–4 experiments).

As we previously reported that angiopoietins activities on neutrophils are Tie2-dependent, we therefore decided to investigate the intracellular pathways downstream of Tie2 activation involved in Ang1-mediated MIP-1β synthesis and release from neutrophils [11, 14, 16]. We and other groups previously reported that depending on the cells being stimulated (endothelial cells or neutrophils), both angiopoietins can mediate their biological activities either through the activation of p38 MAPK, MEK and/or PI3K pathways [10, 14, 15]. In a first set of experiments, we sought to determine whether a p38 MAPK inhibitor (SB203580) or its control vehicle (DMSO; 0.1%) could modulate Ang1-induced MIP-1β synthesis and release. Pretreatment with SB203580 did not significantly affect the basal level of intracellular and extracellular MIP-1β protein content. However, pretreatment with SB203580 (1 and 10 μM) [26] completely prevented the increase of intracellular and extracellular MIP-1β protein content mediated by Ang1 (10 nM) (Fig 2A and 2B). We then assessed by Western blot analyses the effect of Ang1 and SB203580 on p38MAPK phosphorylation. Treatment with Ang1 (10 nM) increased p38MAPK phosphorylation by 4.38-fold (p-p38 / p38 MAPK ratio), which was almost completely abrogated by a pretreatment with SB203580 (10 μM; 91% inhibition) (Fig 2C). As Ang2 was unable to support significant increase of intracellular and extracellular MIP-1β protein content, we assessed its capacity to induce p38MAPK phosphorylation and observed that Ang2 was less potent than Ang1 to promote p38MAPK activation, whereas LPS as positive control was a strong inducer of p38MAPK phosphorylation (Fig 2C).

**Fig 3:**
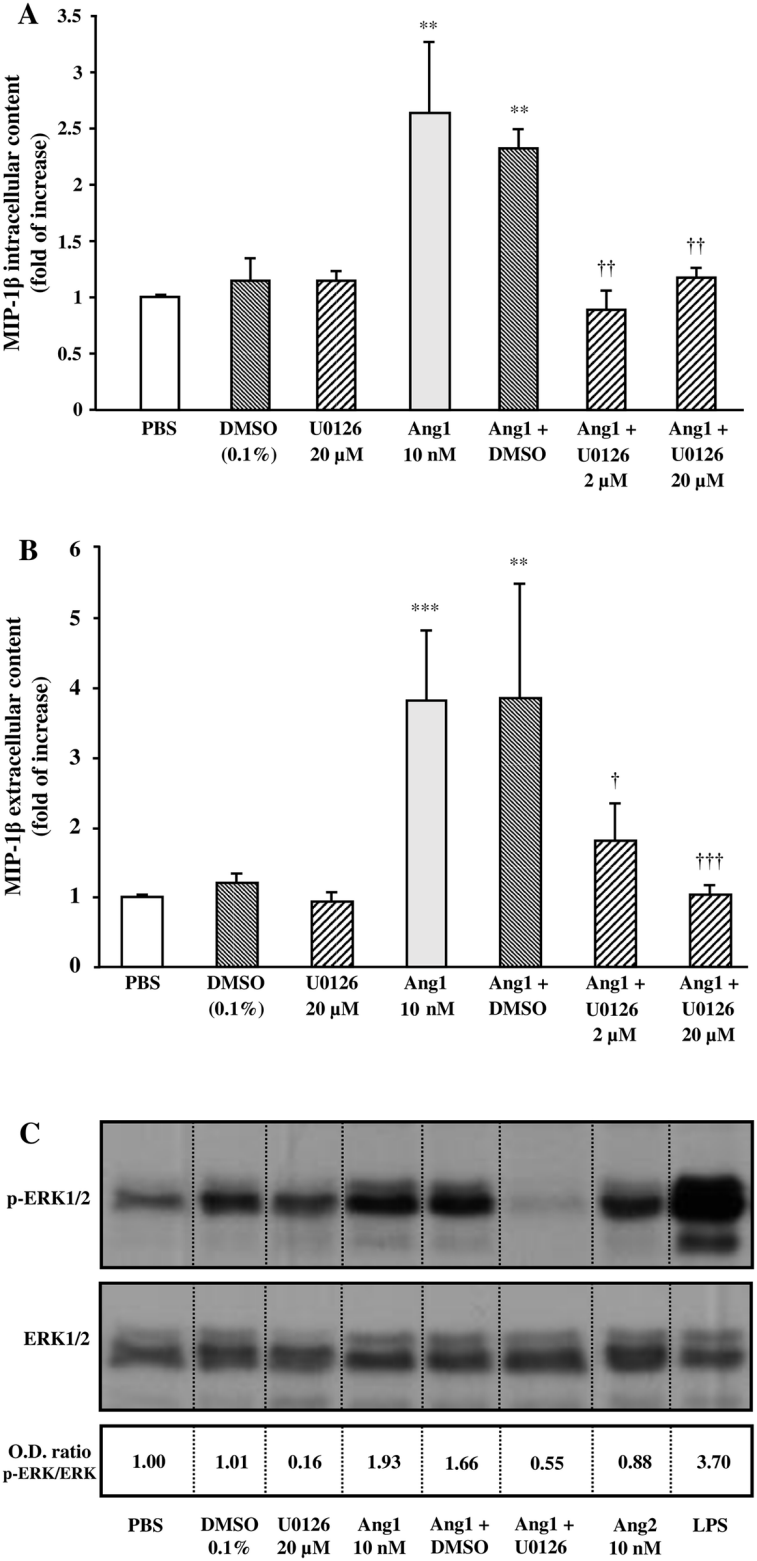
Effect of MEK pathway on Ang1-induced MIP-1β release in neutrophils. Neutrophils were pretreated with or without U0126 (2 or 20 μM) or with DMSO (0.1%; control vehicle) for 5 minutes. Then, neutrophils were incubated with or without Ang1 (10 nM) for 24 hours at 37°C and MIP-1β synthesis (A) and release (B) was evaluated by ELISA. In another set of experiments, neutrophils were stimulated with aforementioned inhibitors ± Ang1 for 2.5 minutes at 37°C; treatment with Ang2 (10 nM) and LPS (100 ng/ml) were added as comparative controls. Cell lysates were collected for Western blot analyses of ERK1/2 phosphorylation and total ERK1/2 protein (C). The image presented is a representative figure of at least three independent experiments. **p<0.01 and ***p<0.001 as compared to PBS-treated cells; ^†^p<0.05, ^††^p<0.01 and ^†††^p<0.001 as compared to Ang1-treated cells (Fig 3A and B; n = 3–4 experiments).

In another set of experiments, we sought to determine whether a selective MEK inhibitor (U0126) could modulate Ang1 effect on MIP-1β synthesis and release. Treatment with U0126 did not modulate basal MIP-1β intracellular and extracellular content. However, pretreatment with U0126 (2 and 20 μM) [27] blocked almost completely Ang1-induced intracellular (107 and 90% inhibition, respectively) and extracellular (71 and 99% inhibition, respectively) MIP-1β protein content (Fig 3A and 3B). By Western blot analyses, we assessed the effect of Ang1 and U0126 on ERK1/2 phosphorylation. Pretreatment with U0126 (20 μM) nearly abrogated basal ERK1/2 phosphorylation. Treatment with Ang1 (10 nM) increased ERK1/2 phosphorylation by 1.93-fold (p-ERK1/2 / ERK1/2 ratio), which was inhibited below the PBS-basal level by a pretreatment with U0126 (Fig 3C). We assessed Ang2 capacity to induce ERK1/2 phosphorylation and observed that Ang2 did not promote ERK1/2 activation, whereas LPS as positive control was a strong inducer of ERK1/2 phosphorylation (Fig 3C).

**Fig 4:**
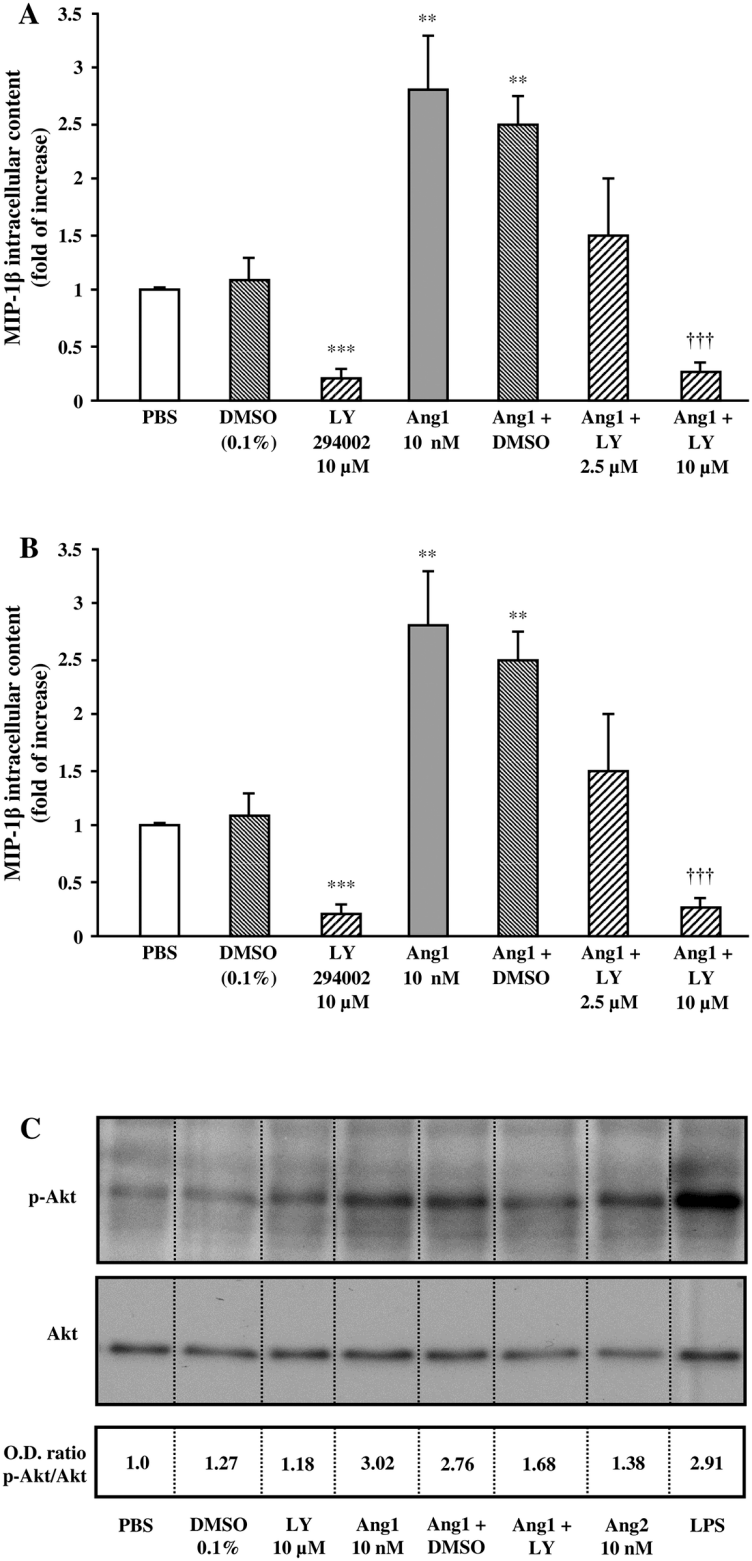
Effect of PI3K pathway on Ang1-induced MIP-1β release in neutrophils. Neutrophils were pretreated with or without LY294002 (2.5 or 10 μM) or with DMSO (0.1%; control vehicle) for 5 minutes. Then, neutrophils were incubated with or without Ang1 (10 nM) for 24 hours at 37°C and MIP-1β synthesis (A) and release (B) was evaluated by ELISA. In another set of experiments, neutrophils were stimulated with aforementioned inhibitors ± Ang1 for 2.5 minutes at 37°C; treatment with Ang2 (10 nM) and LPS (100 ng/ml) were added as comparative controls. Cell lysates were collected for Western blot analyses of PI3K phosphorylation and total PI3K protein (C). The image presented is a representative figure of at least three independent experiments. **p<0.01 and ***p<0.001 as compared to PBS-treated cells; ^†††^p<0.001 as compared to Ang1-treated cells (Figs 4A and B; n = 3–4 experiments).

We next addressed whether a selective PI3K inhibitor (LY294002) could modulate Ang1-induced MIP-1β synthesis and release from neutrophils. Treatment with LY294002 reduced significantly basal MIP-1β intracellular content while having a trend to reduce MIP-1β extracellular content from neutrophils. Moreover, pretreatment with LY294002 (2.5 and 10 μM) [28] prevented the increase of MIP-1β intracellular and extracellular protein level mediated by Ang1 (Fig 4A and 4B). We then looked at PI3K activation by assessing Akt phosphorylation by Western blot analyses. We observed that a treatment with LY294002 (10 μM) reduced basal Akt phosphorylation. Treatment with Ang1 (10 nM) increased Akt phosphorylation by 3.02-fold (p-Akt / Akt ratio), which was inhibited by 72% following a pretreatment with LY294002 (10 μM) (Fig 4C). We assessed Ang2 capacity to induce Akt phosphorylation and observed that Ang2 was less potent than Ang1 to promote Akt activation, whereas LPS as positive control was equivalent to Ang1-mediated Akt phosphorylation (Fig 4C).

### Ang1-mediated MIP-1β synthesis and release is NF-κB-dependent

**Fig 5:**
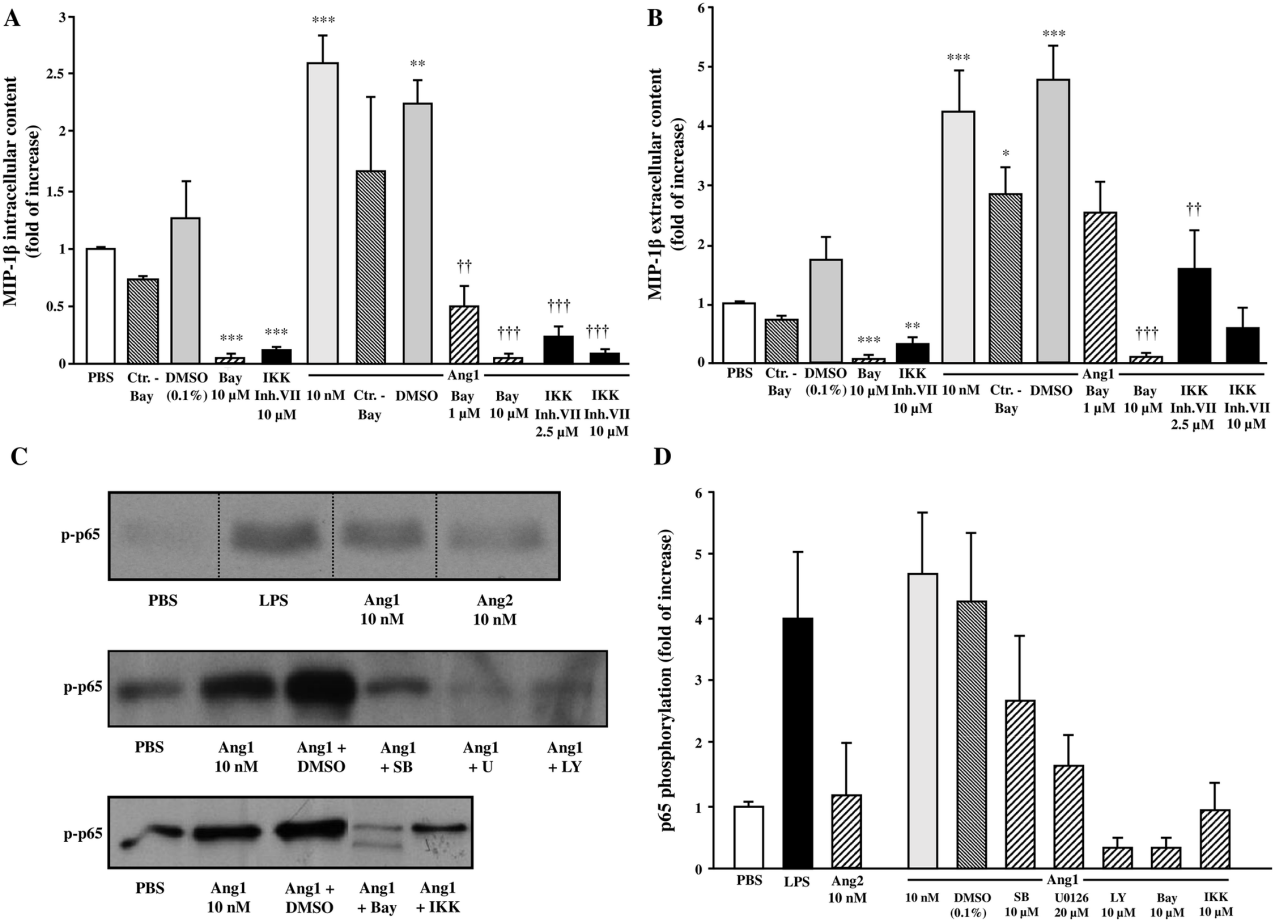
Effect of NF-κB pathway on Ang1-induced MIP-1β release in neutrophils. Neutrophils were pretreated with or without Bay11-7085 (1 or 10 μM) (or with negative control), IKK inhibitor VII (2.5 or 10 μM), SB203580 (10 μM), U0126 (20 μM), LY294002 (10 μM) or with DMSO (0.1%; control vehicle) for 5 minutes. Then, neutrophils were incubated with or without Ang1 (10 nM) for 24 hours at 37°C and MIP-1β synthesis (A) and release (B) was evaluated by ELISA. In another set of experiments, neutrophils were stimulated with aforementioned inhibitors ± Ang1 for 45 minutes at 37°C; treatment with Ang2 (10 nM) and LPS (100 ng/ml) were added as comparative controls. Cell lysates were collected for Western blot analyses of p65 phosphorylation and densitometry analysis (C and D). The image presented is a representative figure of at least three independent experiments. *p<0.05, **p<0.01 and ***p<0.001 as compared to PBS-treated cells; ^††^p<0.01; ^†††^p<0.001 as compared to Ang1-treated cells (Figs 5A and B; n = 4 experiments).

It has been reported that in ECs, Ang1 inhibits VEGF-induced expression of adhesion molecules through modulation of NF-κB [7, 29, 30]. We therefore addressed whether this pathway is involved in Ang1-mediated MIP-1β synthesis and release from neutrophils. In a first set of experiments, we sought to determine whether a NF-κB inhibitor (Bay11-7085) could modulate Ang1-mediated MIP-1β synthesis and release. Treatment with Bay11-7085 reduced significantly the basal MIP-1β intracellular and extracellular content from neutrophils. Likewise, pretreatment with Bay11-7085 (1 and 10 μM) [31] blocked Ang1-induced MIP-1β intracellular (below PBS-mediated basal level) and extracellular (by 47% and below PBS-mediated basal level, respectively) protein content (Fig 5A and 5B). To support these observations, we evaluated the effect of a second NF-κB inhibitor, IKK inhibitor VII, or its corresponding control vehicle (DMSO) on Ang1-mediated MIP-1β synthesis and release from neutrophils. Treatment with IKK inhibitor VII reduced significantly the basal MIP-1β intracellular and extracellular content. Pretreatment with IKK inhibitor VII (2.5 and 10 μM) [32] significantly blocked Ang1-induced MIP-1β intracellular (below PBS-basal level) and extracellular (by 83% and below PBS-mediated basal level, respectively) protein content (Fig 5A and 5B). We then addressed by Western blot analyses the effect of Ang1 on p65 phosphorylation (a subunit of NF-κB) alone and in presence of corresponding inhibitors of the pathways enrolled in Ang1-effect on MIP-1β synthesis and release. Treatment with Ang1 (10 nM) significantly induced p65 phosphorylation (2.3-fold increase), pretreatment with SB203580 (10 μM) reduced Ang1-mediated p65 phosphorylation by 79% inhibition, while the other inhibitors U0126 (20 μM), LY294002 (10 μM), Bay11-7085 (10 μM) and IKK inhibitor VII (10 μM) prevented Ang1-mediated p65 phosphorylation below PBS-mediated basal level (Fig 5C and 5D). We assessed Ang2 capacity to induce p65 phosphorylation and observed that Ang2 was much less potent than Ang1 to promote p65 activation, whereas LPS as positive control was a strong inducer of p65 phosphorylation (Fig 5C and 5D).

### Ang1-induced MIP-1β synthesis and release is transcription- and translation-dependent

**Fig 6:**
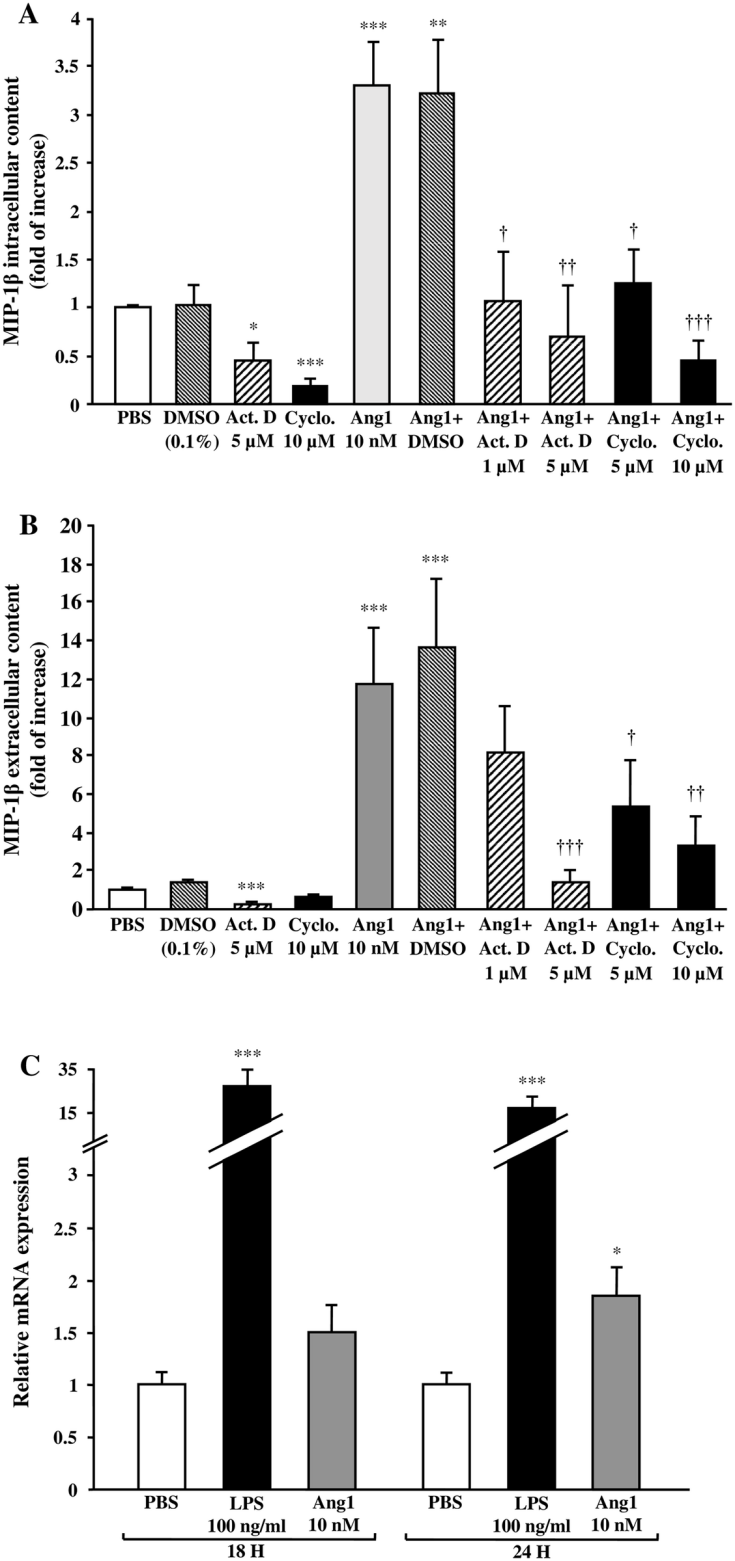
Effect of Ang1 on MIP-1β transcription, translation and mRNA levels in neutrophils. Neutrophils were pretreated with or without actinomycin D (1 or 5 μM), cycloheximide (5 or 10 μM) or with DMSO (0.1%) for 5 minutes. Then, neutrophils were incubated with or without Ang1 (10 nM) for 24 hours at 37°C and MIP-1β synthesis (A) and release (B) were evaluated by ELISA. In another set of experiments, neutrophils were incubated with or without LPS (100 ng/ml) or Ang1 (10 nM) for 18 or 24 hours at 37°C and MIP-1β mRNA was extracted and quantified using RT-qPCR (C). *p<0.05, **p<0.01 and ***p<0.001 as compared to PBS-treated cells; ^†^p<0.05, ^††^p<0.01 and ^†††^p<0.001 as compared to Ang1-treated cells (Figs 6A–C; n = 3–7 experiments).

Since NF-κB is a transcription factor, we addressed whether transcriptional and translational inhibitors (actinomycin D and cycloheximide, respectively) could modulate Ang1 effect on MIP-1β synthesis and release. Treatment with actinomycin D significantly reduced basal MIP-1β intracellular and extracellular content while cycloheximide significantly reduced basal MIP-1β intracellular protein content but had only a trend to reduce basal MIP-1β extracellular protein level (Fig 6A and 6B). Moreover, actinomycin D (1 and 5 μM) [15, 33] completely blocked Ang1-induced MIP-1β intracellular (at or below PBS-basal level) and extracellular (by 52% and below PBS-mediated basal level, respectively) protein content (Fig 6A and 6B). Pretreatment with cycloheximide (5 and 10 μM) [15, 34] also significantly inhibited Ang1-induced MIP-1β intracellular (by 89% and below PBS-mediated basal level, respectively) and extracellular (by 54 and 78% inhibition respectively) protein content (Fig 6A and 6B). To further support these observations, we evaluated the effect of Ang1 on MIP-1β mRNA expression using RT-qPCR technique. Treatment with LPS (positive control; 100 ng/ml) significantly increased MIP-1β mRNA synthesis at 18 and 24 hour (27- and 16-fold of increase, respectively) while Ang1 (10 nM) had a trend to increase MIP-1β mRNA synthesis at 18 hour, which became significant after 24 hours of stimulation (1.5- and 1.8-fold of increase, respectively) (Fig 6C). MIP-1β mRNA synthesis was also evaluated at 1, 2, and 4 hours post-stimulation but Ang1 had no effect (data not shown).

### Effect of intracellular inhibitors on neutrophil viability

We assess the effect of downstream selected inhibitors of p42/44 MAPK (ERK1/2), p38MAPK, PI3K, and NFκB on neutrophil viability by flow cytometry [16]. All the weakest concentrations used in our study were already efficient to reduce in part or completely Ang1-mediated MIP-1ß synthesis and release (intracellular and extracellular protein content) without affecting significantly the neutrophil viability (S1 Fig). At the highest concentrations, all inhibitors were efficient to prevent Ang1-mediated MIP-1ß synthesis and release, and some of them did reduce in part the viability of the neutrophils at 24 hours post-stimulation (S1 Fig).

## Discussion

This study addresses for the first time the capacity of angiopoietins to modulate the expression of MIP-1β mRNA, its protein synthesis and release by the neutrophils. Herein, we report that only Ang1 but not Ang2 is capable to do so. In addition, using selective inhibitors, we delineate that Ang1-induced MIP-1β synthesis and release is p38 MAPK, ERK1/2, PI3K and NF-κB-dependent.

### Differential effects of Ang1 and Ang2 on MIP-1β synthesis and release

We report that only Ang1 has the capacity to induce MIP-1β synthesis and release by the neutrophils while Ang2 has no such effect. Our results are in line with previous studies in which we demonstrated the differential capacity of Ang1 as opposed to Ang2 to induce: 1) IL-1RA mRNA synthesis and protein release, 2) IL-1β and IL-8 mRNA and protein synthesis, and 3) IL-8 release. In addition, we observed that Ang1 prolongs neutrophil viability through IL-8 release, whereas Ang2 has no such effect. Interestingly, all these events require transcriptional and translational activities and were only mediated by Ang1, and at a high concentration (10 nM) [15–17]. To the best of our knowledge, our studies are the only ones reporting transcriptional activities of Ang1 in neutrophils. In ECs, Hussain *et al*. reported that Ang1, in a Tie2-dependent manner, induced AP-1 and Egr-1 transcription factor through the activation of PI3K and MEK pathways [35, 36]. In addition, they reported that Ang1 reduces VEGF-induced leukocyte adhesion to ECs and the expression of adhesion molecules (ICAM-1, VCAM-1 and E-selectin) [37]. The group of Koh also reported the capacity of Ang1 to modulate transcriptional activities in endothelial cells (ECs), and as well, at high concentration (≈3 nM; 200 ng/ml). Namely, the authors reported the capacity of Ang1 to reduce VEGF-mediated expression of adhesion molecules and tissue factor, and a reduction of VEGF-mediated of endothelial cell permeability [7, 23, 38, 39]. On the other hand, recent studies reported the capacity of Ang1 to promote pro-inflammatory activities, namely through its capacity to induce the release of TNF-α by human monocytes [40] and its capacity to activate the innate immune system of the vessel wall, by promoting the release of TNF-α and GRO-1 from cultured aortic rings [41]. On our side, we also reported that Ang1 and Ang2, upon Tie2 activation, can have similar capacity to induce pro-inflammatory activities in ECs and neutrophils. Both angiopoietins can induce endothelial P-selectin translocation, endothelial and neutrophil PAF synthesis, neutrophil β_2_-integrin activation, neutrophil adhesion onto human extracellular matrix (hECM) and activated ECs (HUVECs) as well as neutrophil transmigration in Boyden chamber [10, 11, 14]. It is noteworthy to mention that these effects were independent from transcriptional activities and were achieved at much lower concentrations as low as 1 pM, with maximal activities being achieved at 0.1–1 nM. These observations suggest that at low concentrations (10^−12^–10^−9^ M), Ang1 and Ang2 have equipotent agonistic capacities to promote intracellular signaling pathway activation leading to non-transcriptional-mediated biological activities, while at higher concentrations, only Ang1 is capable to activate distinct signaling pathways leading to transcriptional and translational activities associated either to the repression or induction of pro-inflammatory cytokines expression. Therefore, considering that Ang1 and Ang2 have similar protein structure (60% of homology) and binding affinity for Tie2 receptor [42, 43], these results suggest that Ang2 acts as a biased agonist [44, 45], with maximal full agonistic activity as compared to Ang1 at lower concentrations, and having no or minor capacity at needed higher concentration to promote transcriptional and translational activities leading to selected protein mRNA expression, protein synthesis and/or release. In addition, using a RT-qPCR array to assess the modulation of 84 genes expression involved in inflammatory response, we reported that only Ang1 was able to significantly modulate gene expression of specific inflammatory cytokines and their receptors in neutrophils while Ang2 had no significant effect on any of those genes [17].

### Ang1 induces MIP-1β synthesis and release in neutrophils after 24 hours of stimulation

Neutrophils are the first innate leukocytes being recruited at inflamed/injured sites to promote a wide range of pro-inflammatory activities (e.g. respiratory burst, cellular degranulation, release of inflammatory cytokines, metalloproteinase (MMPs), neutrophil extracellular traps (NETs), microparticles and growth factors) to induce cellular toxicity to kill pathogens and to attract other leukocytes [18, 46]. In that context, angiopoietins induce acute non-transcriptional activities (PAF synthesis, β_2_-integrin translocation, neutrophil adhesion and transmigration), which are achieved within 0–60 minutes post-stimulation. In addition, Ang1 also promotes acute transcriptional activities in neutrophils. Namely, Ang1-induced IL-8 synthesis and release are plateauing within 2 hour post-stimulation, while IL-1RA release is optimal by 4 hours post-stimulation [11, 14, 15, 17]. In contrast, it is interesting to note that Ang1-induced MIP-1β synthesis and release (intracellular and extracellular MIP-1β protein content) is delayed, starting at 18 hours post-stimulation and being statistically significant only after 24 hours of treatment. This late effect could have been related to Ang1 capacity to prolong neutrophil viability, thus, we included in our study other pro-survival agonists (e.g. IL-8 and PAF). Since Ang1 and IL-8 have a similar capacity to increase MIP-1β intracellular and extracellular protein levels; whereas PAF has no such effect, it is suggesting that the increase of neutrophil viability is not associated to an increase of intracellular and extracellular MIP-1β protein content.

Taken into account the time-dependent biological activities mediated by Ang1 in neutrophils, and now including the late MIP-1β synthesis and release, our data suggest a well structured and coordinated *sequelae* of events mediated by Ang1 in neutrophils. First, Ang1-stimulated neutrophils are quickly transmigrating toward tissues expressing chemotactic cytokines including Ang1, which can be released by SMCs, platelets, pericytes and neutrophils themselves under specific stimuli [14, 21, 42, 47]. Once at these angiogenic/inflammatory sites, neutrophils secrete acute pro-inflammatory mediators, such as PAF and IL-8, to sustain inflammation and recruit leukocytes, mainly neutrophils through IL-8 [11, 15]. Ang1-stimulated neutrophils can also secrete anti-inflammatory mediators; namely, IL-1RA after 4 hours of stimulation, supporting the mounting evidences that neutrophils can also counteract their damaging activities upon their first activation phase [17]. Indeed, depending on the degree of inflammation and the balance of pro- and anti-inflammatory mediators at specific Ang1-expressing sites, neutrophils can display a pro- or an anti-inflammatory profile [48, 49]. The late release of MIP-1β would then induce a significant MΦ efflux [20]. This late entry of the monocytes could allow them to initiate distinct differentiation pathways into either M2-macrophages, inducing the release of anti-inflammatory mediators (e.g. IL-10) and promoting the resolution of inflammation, or into M1-macrophages, inducing the release of pro-inflammatory mediators (e.g. IL-1β, IL-12 and TNF-α), and thereby, sustaining the pro-inflammatory response [50]. Therefore, the late MΦ efflux generated by Ang1-induced MIP-1β release could allow the resolution of inflammation in physiological conditions while supporting chronic inflammation and vascular pathologies in a pathological environment. Indeed, it has been reported that in diseases such as rheumatoid arthritis and solid tumors, abundance of leukocytes, mainly neutrophils and MΦ, promote pathological angiogenesis that further support the inflammatory response, creating a vicious cycle [51].

In our study, we observed that compared to Ang1, LPS is a powerful inducer of MIP-1β (CCL4) synthesis and release and our data are in-line with a recent report [22]. However, when we compared Ang1 to other pro-inflammatory agonists, namely IL-8 and PAF [16, 24, 25], we observed that Ang1 is as powerful as IL-8 to induce MIP-1β synthesis and release, whereas PAF has no significant effect. Our study is also in agreement with a recent study reporting that LPS induces the synthesis and release of MIP-1β with maximal effect being achieved between 6 to 12 hours [22]. One possible explanation why LPS is much more potent and faster than other agonists to promote MIP-1β synthesis and release could be due to the fact that LPS is a bacterial membrane component, and it is essential that neutrophils react rapidly and strongly to this pathological inflammatory mediator to stop any infection. Therefore, it is logical that LPS induced a quicker and stronger response toward MIP-1β intracellular and extracellular levels as compared to non-infectious related inflammatory mediators such as IL-8 and Ang1. It is also to mention that in function of the cytokines to be produced, Ang1 can also promote earlier transcriptional and translational activities, for instance, we reported that in human neutrophils, Ang1 is leading up to a 5-fold increase of IL-8 and IL-1β synthesis within 1 hour and peaking within 2 and 6 hours respectively [15, 17]. Our studies demonstrate that in function of the cytokines being expressed, Ang1 is capable to promote a programmed acute and delayed synthesis and release of pro-inflammatory mediators by human neutrophils.

### Intracellular mechanisms involved in Ang1-mediated MIP-1β synthesis and release

In previous studies, we detailed the mechanisms by which LPS and Ang1 mediated IL-8 and IL-1ß synthesis and release by human neutrophils. In both cases we observed that Ang1 mediated IL-8 synthesis and release was p42/44 MAPK-dependent, whereas LPS-mediated IL-8 synthesis and release was p38MAPK-dependent. Similarly Ang1- and LPS-mediated IL-1ß synthesis was p42/44 MAPK-dependent and p38MAPK-dependent respectively. It has previously been detailed that LPS mediates human neutrophil MIP-1ß synthesis and release through the activation of p38MAPK, p42/44 MAPK, PI3K and downstream NFκB activation [52, 53]. In the current study, it is interesting to observe that all 3 (p38MAPK, p42/44 MAPK and PI3K) kinases are also involved in Ang1-mediated MIP-1ß synthesis and release. As the 3 kinases are involved, as opposed to Ang1-mediated IL-8 or IL-1ß synthesis and release, which were only p42/44 MAPK-dependent it might also provide a potential explanation why MIP-1ß synthesis and release imply a longer expression profile. In addition, although these 3 kinases were rapidly activated (within minutes) and leading to rapid downstream activation of NFκB either by LPS or Ang1, we cannot explain at this time, why the processing of MIP-1ß synthesis and release is observed at a later time point as compared to the expression profile of other pro-inflammatory cytokines.

It was reported that in ECs, Ang1 inhibits VEGF-induced expression of adhesion molecules through PI3K activation, which interferes with VEGF-mediated NF-κB activation [7]. The link between Ang1 and NF-κB in ECs was further supported by the group of Brindle who reported that upon Tie2 phosphorylation, ABIN-2 (A20 binding inhibitor of NF-κB activation-2) associates with Tie2 and that this interaction is responsible for Ang1 inhibitory effects on NF-κB signaling [29, 30]. Taken together, these results suggest that Ang1 inhibits pro-inflammatory NF-κB transcriptional signaling activities in ECs while having opposite transcriptional activity in neutrophils. This differential behavior is in line with previous reports demonstrating that in function of cell types, NF-κB can be differentially activated [54, 55]. Our study also demonstrating that p38 MAPK, MEK and PI3K pathways are all essential to Ang1-related NF-κB phophorylation, as the inhibitors of these pathways were all capable to reduce or to obliterate p65 phosphorylation. These latter observations are also supported by other studies reporting that p38 MAPK, MEK and PI3K can regulate NF-κB activation in neutrophils and/or other cell types [52, 53, 56, 57].

**Fig 7:**
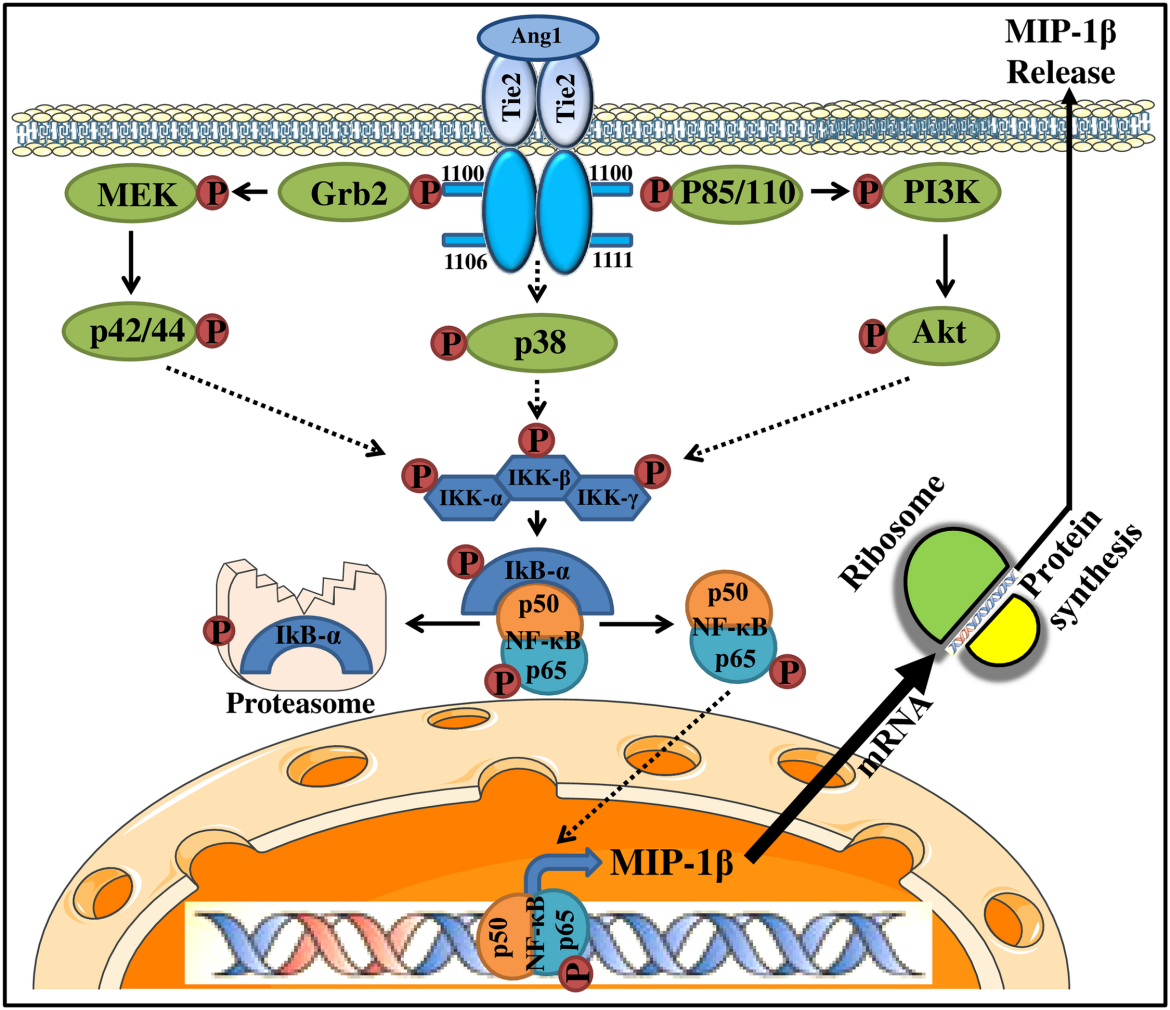
Proposed intracellular signaling pathways involved in Ang1-mediated MIP-1β synthesis and release in human neutrophils.

In summary, we report for the first time the differential capacity of angiopoietins to promote transcriptionnal activities in neutrophils. Namely, Ang1, at high concentration, supports late transcriptional and translational activities associated to MIP-1β synthesis and release through NF-κB activation, while Ang2 has no effect on these activities. Moreover, Ang1-induced MIP-1β protein synthesis and release are regulated through p38 MAPK, MEK and PI3K pathways and subsequent downstream NF-κB activation (Fig 7). These results provide further evidence of the implication of Ang1 in promoting pro-inflammatory activities through the activation of neutrophils, which in turn, can promote the recruitment of other leukocytes namely MΦ through their capacity to promote the synthesis and release of MIP-1β.

## Supporting Information

S1 Fig Effect of inhibitors on neutrophil viability. Human neutrophils (5 x 10^6^ / mL) were incubated for 24 hours with PBS, DMSO 0.1% (control vehicle), SB203580 (1 and 10 μM), U0126 (2 and 20 μM), LY294002 (2.5 and 10 μM), BAY 11–7085 (1 and 10 μM) and IKK Inhibitor VII (2.5 and 10 μM). Neutrophil viability was then measured by flow cytometry following a 20 minutes neutrophil incubatin with annexin-V-FITC (5 uL) and propidium iodide (PI; 10 uL) (Becton Dickinson) [16]. Neutrophils were considered viable in the absence of positive staining for both markers (annexin-V and P.I.; annexin-V^−^/P.I.^−^). *p < 0.05 and **p < 0.01 as compared to PBS-treated cells. (n = 6–8 experiments per condition).
(TIF)
