# Supplementary figures and images for: New Insights into the Pro-Inflammatory Activities of Ang1 on Neutrophils: Induction of MIP-1β Synthesis and Release
Source: PLoS One. 2016 Sep 15;11(9):e0163140. doi: 10.1371/journal.pone.0163140 (PMC5025150; doi:10.1371/journal.pone.0163140)

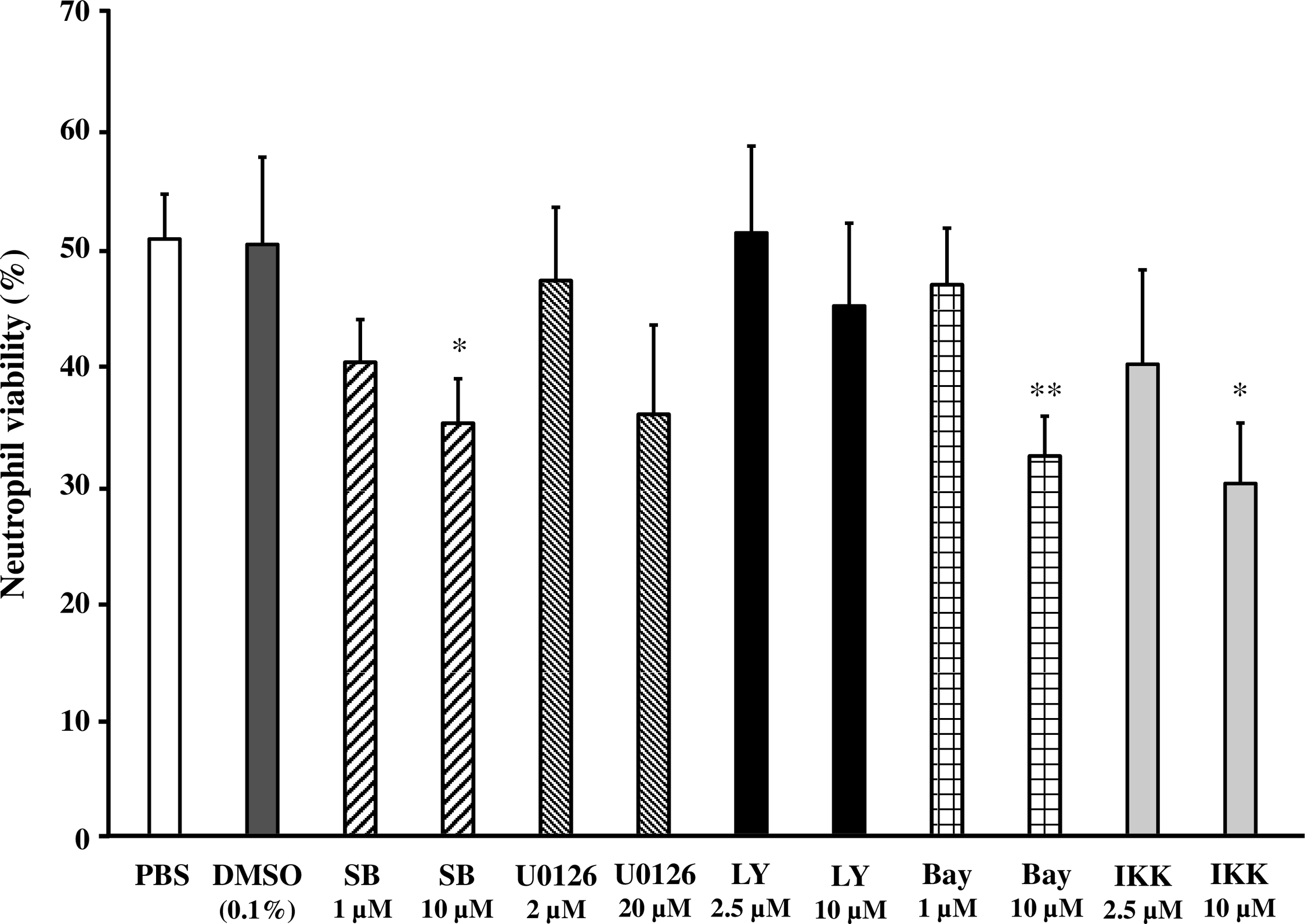

Supplement: S1 Fig — Human neutrophils (5 x 10^6^ / mL) were incubated for 24 hours with PBS, DMSO 0.1% (control vehicle), SB203580 (1 and 10 μM), U0126 (2 and 20 μM), LY294002 (2.5 and 10 μM), BAY 11–7085 (1 and 10 μM) and IKK Inhibitor VII (2.5 and 10 μM). Neutrophil viability was then measured by flow cytometry following a 20 minutes neutrophil incubatin with annexin-V-FITC (5 uL) and propidium iodide (PI; 10 uL) (Becton Dickinson) [16]. Neutrophils were considered viable in the absence of positive staining for both markers (annexin-V and P.I.; annexin-V−/P.I.−). *p < 0.05 and **p < 0.01 as compared to PBS-treated cells. (n = 6–8 experiments per condition). (TIF) [file pone.0163140.s001.tif]
